# Identification and characterization of in vivo, in vitro and reactive metabolites of vandetanib using LC–ESI–MS/MS

**DOI:** 10.1186/s13065-018-0467-5

**Published:** 2018-09-24

**Authors:** Mohamed W. Attwa, Adnan A. Kadi, Hany W. Darwish, Sawsan M. Amer, Nasser S. Al-shakliah

**Affiliations:** 1 Department of Pharmaceutical Chemistry, College of Pharmacy, King Saud University, P.O. Box 2457, Riyadh, 11451 Kingdom of Saudi Arabia; 2 Analytical Chemistry Department, Faculty of Pharmacy, Cairo University, Kasr El-Aini St, Cairo, 11562 Egypt

**Keywords:** *N*-methyl piperidine, Vandetanib, In vivo metabolites, In vitro metabolites, Cyano conjugates

## Abstract

Vandetanib (Caprelsa tablets, VNT) is an orally inhibitor of vascular endothelial growth factor receptor 2. The current research reports the characterization and identification of in vitro, in vivo and reactive intermediates of VNT. In vitro metabolites of VNT were performed by incubation with rat liver microsomes (RLMs). Extraction of vandetanib and its in vitro metabolites from the incubation mixtures were done by protein precipitation. In vivo metabolism was done by giving one oral dose of vandetanib (30.8 mg/kg) to Sprague Dawley rats in metabolic cages by using oral gavage. Urine was gathered then filtered at certain time intervals (0, 6, 12, 18, 24, 48, 72, 96 and 120 h) from vandetanib dosing. A similar volume of ACN was added to each collected urine sample. Both layers (organic and aqueous) were injected into liquid chromatography electro spray ionization tandem mass spectrometry (LC–ESI–MS/MS) to detect in vivo vandetanib metabolites. *N*-methyl piperidine ring of vandetanib is considered a cyclic tertiary amine that undergoes metabolism forming iminium intermediates that are very reactive toward nucleophilic macromolecules. Incubation of vandetanib with RLMs in the presence of 1.0 mM KCN was made to check reactive metabolites as it is usually responsible for noticeable idiosyncratic toxicities including phototoxicity and QT interval prolongation. Four in vivo phase I, one in vivo phase II metabolites, six in vitro phase I metabolites and four cyano conjugates of vandetanib were detected by LC–MS/MS. In vitro and in vivo phase I metabolic reactions were *N*-oxide formation, *N*-demethylation, α-carbonyl formation and α-hydroxylation. In vivo phase II metabolic reaction was direct conjugation of vandetanib with glucuronic acid. All metabolic reactions occurred in *N*-methyl piperidine of vandetanib which causes toxicity and instability of vandetanib.

**Electronic supplementary material:**

The online version of this article (10.1186/s13065-018-0467-5) contains supplementary material, which is available to authorized users.

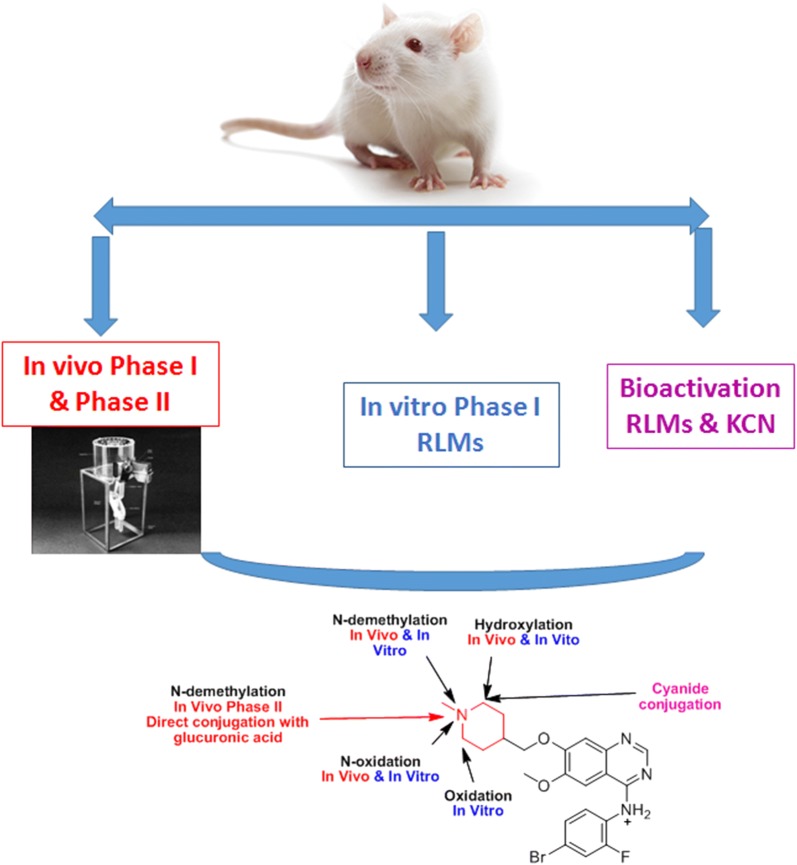

## Introduction

Vandetanib (ZD6474) is an available orally inhibitor of vascular endothelial growth factor receptor 2 (VEGFR) [1]. VEGFR has gained great importance as pharmacologic targets as a Tyrosine kinase receptors [2]. Vandetanib, on 6 April 2011, was approved by the FDA for the treatment of patients suffered from symptomatic or progressive medullary thyroid cancer with unresectable, locally advanced, or metastatic disease. It was considered the first drug approved for this case. The trade name of vandetanib was Caprelsa tablets (AstraZeneca Pharmaceuticals LP). Sudden death and QT prolongation of the are severe side effects for vandetanib [3].

Metabolism is detoxification process of xenobiotics and endogenous compounds by transforming into more hydrophilic compounds to allow excretion outside the body. Drug metabolism work is an essential step in the process of drug discovery, and is usually the factor that evaluate the degree of given drug success to take the approval and to reach the market [4]. Drug metabolism research is done through in vitro and in vivo techniques. In vivo metabolism was performed through the single dose administration of vandetanib to rat using oral gavage followed by gathering of urine samples, at specific time intervals, that contain the drugs and their possible metabolites. In vitro techniques include drugs incubation with various types of in vitro preparations (e.g. hepatocytes and liver microsomes) separated from rats then sample processing and analysis using chromatographic techniques.

Phase I metabolism either in vitro or in vivo of cyclic tertiary amines generates oxidative metabolites including: α-carbonyl formation, ring opening metabolites, *N*-oxygenation, ring hydroxylation and *N*-dealkylation. Metabolites are often less toxic than parent molecules, but occasionally undergo bioactivation forming unstable reactive intermediates that considered more toxic in comparison to parent molecules [5–7]. Reactive metabolites can covalently bind to proteins, which is considered the initiating step in the process of drug-induced organ toxicities [8, 9].

*N*-methyl piperidine ring is a part of vandetanib chemical structure that is considered a cyclic tertiary amine. Drugs that contain cyclic tertiary amine group are able to form iminium intermediates which are hard nucleophiles [10–12]. GSH or its derivatives are not the appropriate as capturing agent for hard nucleophiles while potassium cyanide (KCN) is the best agent for trapping these reactive intermediate including iminium ion _ENREF_7 [5] resulted in stable adducts formation which can be characterized, separated and detected using LC–MS/MS [13, 14].

Since bioactivation is often considered the central reason for observed side effects including phototoxicity and prolongation of QT interval [3, 15], we tested the reactive metabolites formation by incubation of vandetanib with 1.0 mM KCN. Upon literature review, *N*-demethyl vandetanib, vandetanib *N*-oxide and glucuronide conjugate were found in plasma, urine, and feces [1]. The full mechanism of bioactivation of vandetanib is not yet reported.

## Chemicals and methods

### Chemicals

All chemicals are mentioned in Table 1. Rat liver microsomes (RLMs) were prepared in house according to previously published protocol [16–20].

**Table 1:** List of chemicals and materials

Name^a^	Source
Dacomitinib	LC Laboratories (MA, USA)
Acetonitrile (ACN, HPLC-grade), ammonium formate (NH_4_COOH), poly ethylene glycol 300 (PEG 300), dimethyl sulfoxide (DMSO), potassium cyanide (KCN) and formic acid (HCOOH)	Sigma-Aldrich (USA)
Tween 80	Eurostar Scientific Ltd. (Liverpool, UK)
Water (HPLC grade)	Milli-Q plus purification instrument (USA)
Sprague–Dawley rats^b^	The experimental animal care center at King Saud University (KSA)

^a^ All reference powders are of analytical grade and solvent are of HPLC grade

^b^ The University’s Ethics Review Committee approved the animal experimental design

### RLMs incubations

Vandetanib (20 µmol/mL) was incubated at with RLMs (1.0 mg/mL), NADPH (1.0 mmol/mL) and K/Na phosphate buffer (50 mmol/mL, pH 7.4) containing MgCl_2_ (3.3 mmol/mL). Incubation was done at thermostatted shaking water bath (37 °C) for 60 min before the reactions were quenched using two mL of ACN (ice-cold). The incubation mixtures were centrifuged at 14,000 rpm for 12 min then the supernatants were collected then subjected to dryness under a stream of N_2_. Samples residues were reconstituted in mobile phase (95% solvent A and 5% solvent B). The same steps were repeated using a trapping agent (KCN at 1.0 mmol/mL) to capture reactive intermediates forming adducts.

### In vivo metabolism of vandetanib

Six male Sprague–Dawley rats of average weight (340 g) and 4 weeks of age were brought from animal house of King Saud University (Riyadh, KSA). Each rat was housed in special metabolism cage that was placed in animal care facility in a 12-h light/dark cycle (7:00–19:00). Rats had free access to standard water and animal food. Rats were maintained in metabolism cages for 72 h before study starting. Vandetanib was formulated in special solution (5% Tween 80, 4% DMSO, 30% PEG 300, HPLC H_2_O) to allow dispersion of vandetanib. Each rat received a calculated vandetanib depending on its weight.

The Recommended vandetanib dose is 300 mg per day until unacceptable toxicity or disease progression occurs. Average vandetanib dose for human is 5 mg/kg. Rat dose was calculated using these equations [21–23]:

$$ {\text{Rat }} ( {\text{mg}}/{\text{kg}} ) = {\text{Human }}( {\text{mg}}/{\text{kg}} ) * {\text{Human Km}} / {\text{Rat Km}}  $$

$$ {\text{Rat }}( {\text{mg}}/{\text{kg}} ) = 5*{37/6} $$

$$ {\text{Rat }}( {\text{mg}}/{\text{kg}} ) = 185/6 $$

$$ {\text{Rat }}( {\text{mg}}/{\text{kg}} ) = 30.8\;( {\text{mg}}/{\text{kg}} )$$

So the dose for rat was 30.8 mg/kg. Rats were given a single calculated dose of vandetanib. One rat was used as a control and was given solvent without vandetanib. Oral gavage was used for vandetanib dosing to rats. Urine samples were collected after draining into compartments fixed to metabolism cages before vandetanib dosing as control sample and at specific time periods (6, 12, 18, 24, 48, 72, 96 and 120 h) following vandetanib dosing. Filtration of Urine samples was done using 0.45 µm syringe filters for discarding of particulate matters in the urine. A similar volume of ACN was added to each collected urine sample and then the resulted mixture was shaken by vortexing for 1 min. After storing the mixture at 4 °C overnight, two solvent layers (upper organic layer and lower aqueous layer) were formed. Both layers were evaporated until dryness under stream of N_2_ and reconstituted respectively in 1 mL of mobile phase and transferred to HPLC Agilent vials for LC–MS/MS analysis. Control urine samples obtained from rats before drug dosing were done in the same way described for sample purification method. These samples were analyzed by LC–MS/MS to obtain control chromatograms.

### Chromatographic conditions

The adjusted liquid and mass chromatographic conditions for the separation and identification of in vitro and in vivo vandetanib metabolites are described in details in Table 2.

**Table 2:** Optimized parameters of the established LC–MS/MS methodology

LC parameters				MS/MS parameters		
HPLC	Agilent 1200 (Agilent Technologies, CA, USA)			Mass spectrometer	Agilent 6410 QqQ (Agilent Technologies, CA, USA)	
Mobile phase (gradient)	Aqueous phase: 10 mM Ammonium formate in H_2_O (pH: 4.1 using Formic acid)			Ionization source	Positive electrospray ionization (ESI)	
	Organic phase: ACN (0.1% Formic acid)				Drying gas: N_2_ gas Pressure (55 psi) Flow rate (12 L/min)	
	Flow rate: 0.2 mL/min					
	Elution time: 90 min					
Agilent eclipse plus C_18_ Column		In vitro	In vivo		ESI temperature: 350 °C	
	Length (mm)	50	150		Capillary voltage: 4000 V	
	Internal diameter (mm)	2.1	2.1	Collision gas	N_2_ (high purity)	
	Particle size (μm)	1.8	3.5	Modes	Product ion (PI) and full mass scan and	
	Temperature:	22 ± 2 °C		Software	Mass Hunter software	
Elution system	Time (min)	%B (ACN)		Analyte	Vandetanib, in vivo, in vitro and reactive metabolites	
	0	5				
	60	25		Mass conditions	Fragmentor voltage (V)	140
	80	70				
	90	5				
	Post time 15	5			Collision energy (eV)	15

### Identification of in vitro metabolites, in vivo metabolites and cyano conjugates of vandetanib

Extracted ion chromatograms (EICs) for the vandetanib proposed metabolites were used to identify metabolites in the total ion chromatogram (TIC) of ether RLMs incubation extract or urine extract. CID of proposed metabolites molecular ion peaks (MIP) of was performed in the collision cell to get product ion (PI) mass spectra. Structures of metabolites were done by reconstructing the product ions. In vivo vandetanib-related metabolites were concentrated in the organic layer while endogenous components of the urine and highly polar metabolites were located in the aqueous layer.

## Results and discussion

### Identification of in vitro phase 1 vandetanib metabolites

Six phase 1 metabolites were identified: one demethylated (*m/z *− 14) which was identified as VA461, two metabolites with one *N*-oxide or mono hydroxyl (*m/z *+ 16) which were identified as VA491a and VA491b, one metabolite with oxidation of α-carbon and *N*-demethylation of *N*-piperidine which was identified as VA475 and two metabolites at *m/z* 489 which was identified as VA489a and VA489b (Table 3). Six metabolites were formed by incubation of vandetanib with RLMs through four metabolic reactions: *N*-demethylation, *N*-oxide formation, α-carbonyl formation, and α-hydroxylation (Table 3).

**Table 3:** Phase I metabolites of Vandetanib using MS scan and PI scan

	MS scan	Major daughter ions	t_R_ (min)	Metabolic pathway	Proposed conjugate composition	Previously detected (reference)
VNT	475	112	50.4		V + H	
VA461	461	364, 98	49.7	*N*-demethylation	V − CH_2_ + H	+ [1]
VA475	475	112, 110	54.7	*N*-demethylation and α oxidation	V − CH_2_ + O + H	
VA489a	489	126	66.8	α oxidation	V + O − 2H + H	
VA489b	489	364	67.9	*N*-demethylation and 2 α oxidation	V − CH_2_ + 2O + H	
VA491a	491	128, 111	57.1	α Hydroxylation	V + O + H	
VA491b	491	189, 128	50.4	*N*-oxidation	V + O + H	+ [1]

#### Identification of vandetanib and VA475 metabolite

**Fig. 1:**
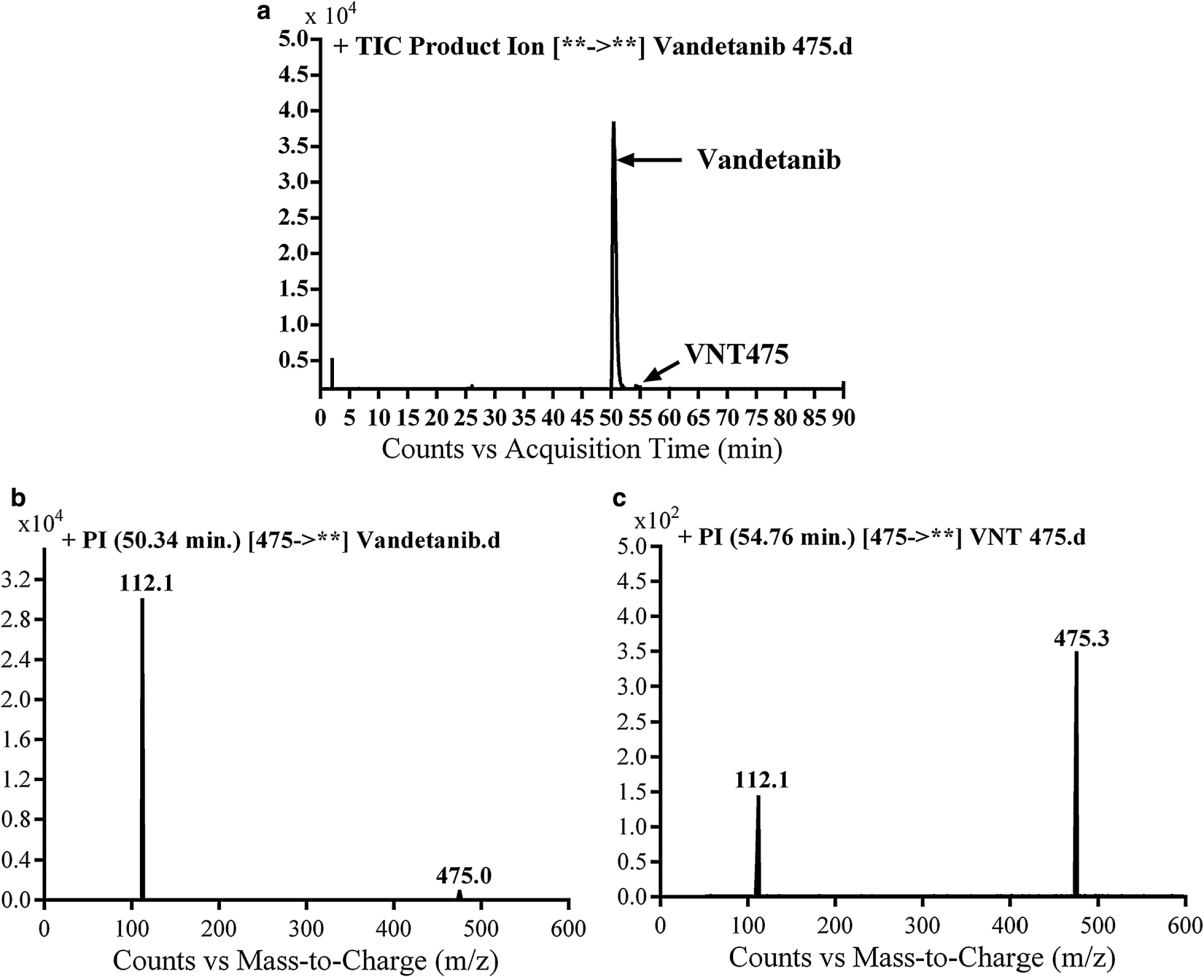
EIC of MIP at *m/z* 475 showing two peaks; vandetanib (50.3 min) and VA475 (54.8 min) (**a**), PI mass spectrum of vandetanib (**b**) and PI mass spectrum of VA475 at *m/z* 475 (**c**)

**Scheme 1:**
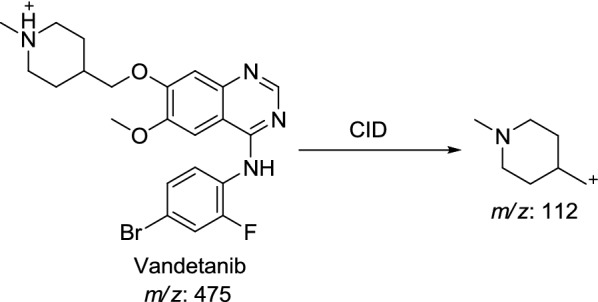
Proposed CID of vandetanib

Vandetanib and VA475 metabolite MIPs were detected at *m/z* 475 in full MS scan mode at retention times (t_R_) of 50.3 and 54.7 min, respectively (Fig. 1a). Upon CID of MIPs at *m/z* 475 gave different daughter ions (Fig. 1b). Collision induced dissociation (CID) of vandetanib inside collision cell of triple quadruploe at *m/z* 475 provided one daughter at *m/z* 112 (Fig. 1b). Daughter at *m/z* 112 represents methyl *N*-methyl piperidine moiety (Scheme 1).

**Scheme 2:**
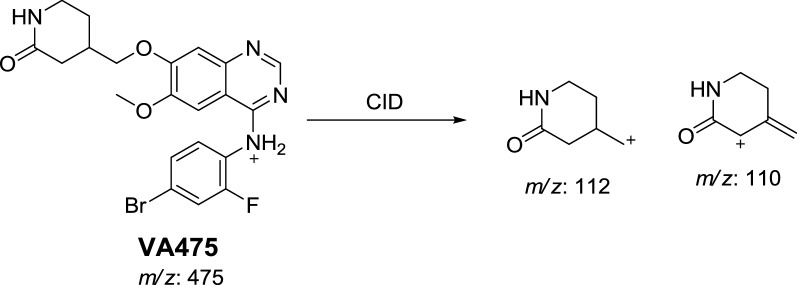
Proposed CID of VA475

CID of VA475 MIP at *m/z* 475 gave daughters at *m/z* 112 and 110 in PI scan by QqQ MS (Fig. 1c). The fragment ion at *m/z* 112 proposed the removal of the methyl group from the *N*-methyl piperidine and oxidation of α-carbon in the ring which matched with the other daughter ion at *m/z* of 110 (Scheme 2).

#### Identification of VA461 metabolite

**Fig. 2:**
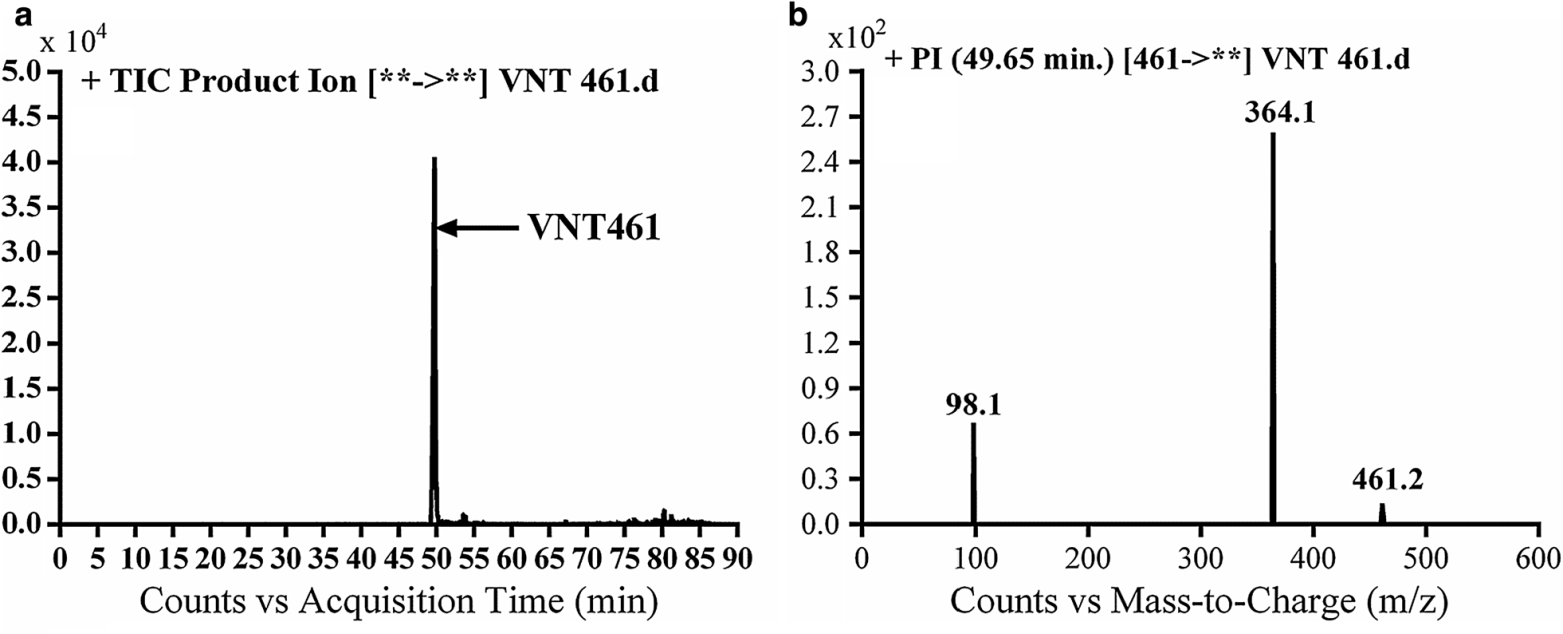
EIC of MIP at *m/z* 461 showing one peak (VA461) at 49.7 min (**a**) and PI mass spectrum of VA461 at *m/z* 461 (**b**)

**Scheme 3:**
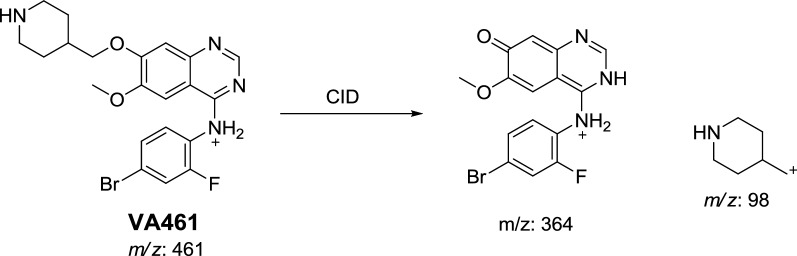
Proposed CID of VA461

VA461 metabolite of Vandetanib was detected at *m/z* 461 in full scan mode at t_R_ of 49.7 min (Fig. 2a). CID of MIP at *m/z* 461 generates fragment ion at *m/z* 98 (Fig. 2b). The daughter ion at *m/z* 98 supposed that the metabolic pathway is *N*-demethylation of the methyl group from the methyl piperidine ring, which matched with the other fragment ions at *m/z* 364. VA461 metabolite was the net product of removal of methyl group from *N*-methyl piperidine group in vandetanib (Scheme 3).

#### Identification of VA489 metabolite

**Fig. 3:**
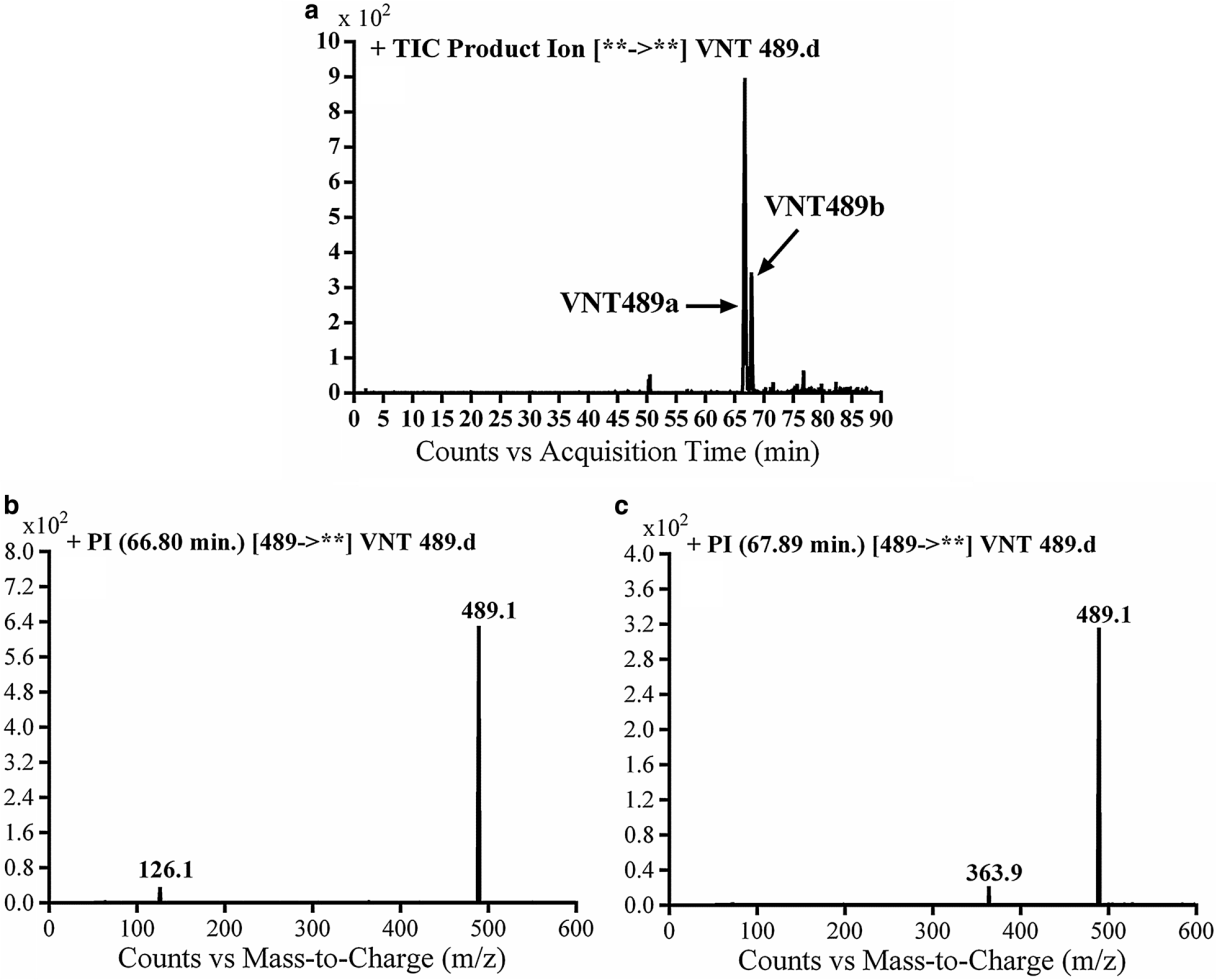
EIC of MIP at *m/z* 489 showing two peaks: VA489a (66.8 min) and VA489b (67.9 min) (**a**), PI mass spectra of VA489a (**b**) and VA489b at *m/z* 489 (**c**)

VA489a and VA489b metabolites of vandetanib were detected at *m/z* 489 in MS scan mode at t_R_ of 66.8 and 67.9 min, respectively (Fig. 3a). CID of MIPs at *m/z* 489 gave various daughter ions (Fig. 3b, c).

**Scheme 4:**
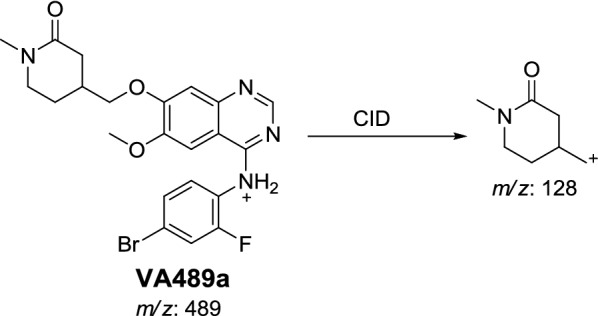
Proposed CID of VA489a

In case of VA489a, the fragment ion at *m/z* 126 supposed that the metabolic reactions were α-carbonyl formation of *N*-methyl piperidine group (Scheme 4).

**Scheme 5:**
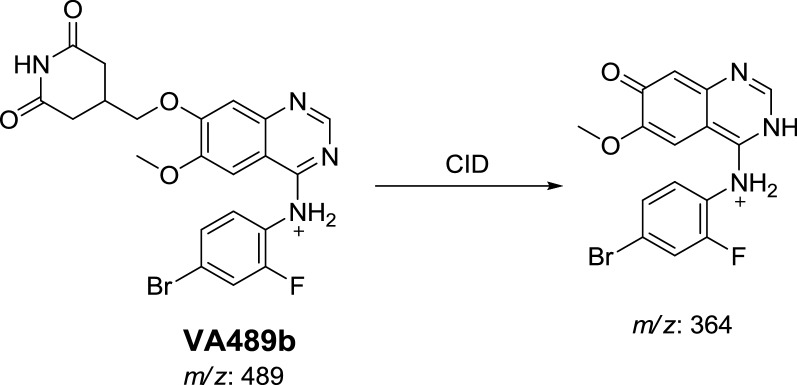
Proposed CID of VA489b

In case of VA489a, the fragment ion at *m/z* 364 supposed that metabolic reactions were 2 α-carbonyl formation and *N*-demethylation at *N*-methyl piperidine ring (Scheme 5).

#### Identification of VA91a and VA491b metabolite

**Fig. 4:**
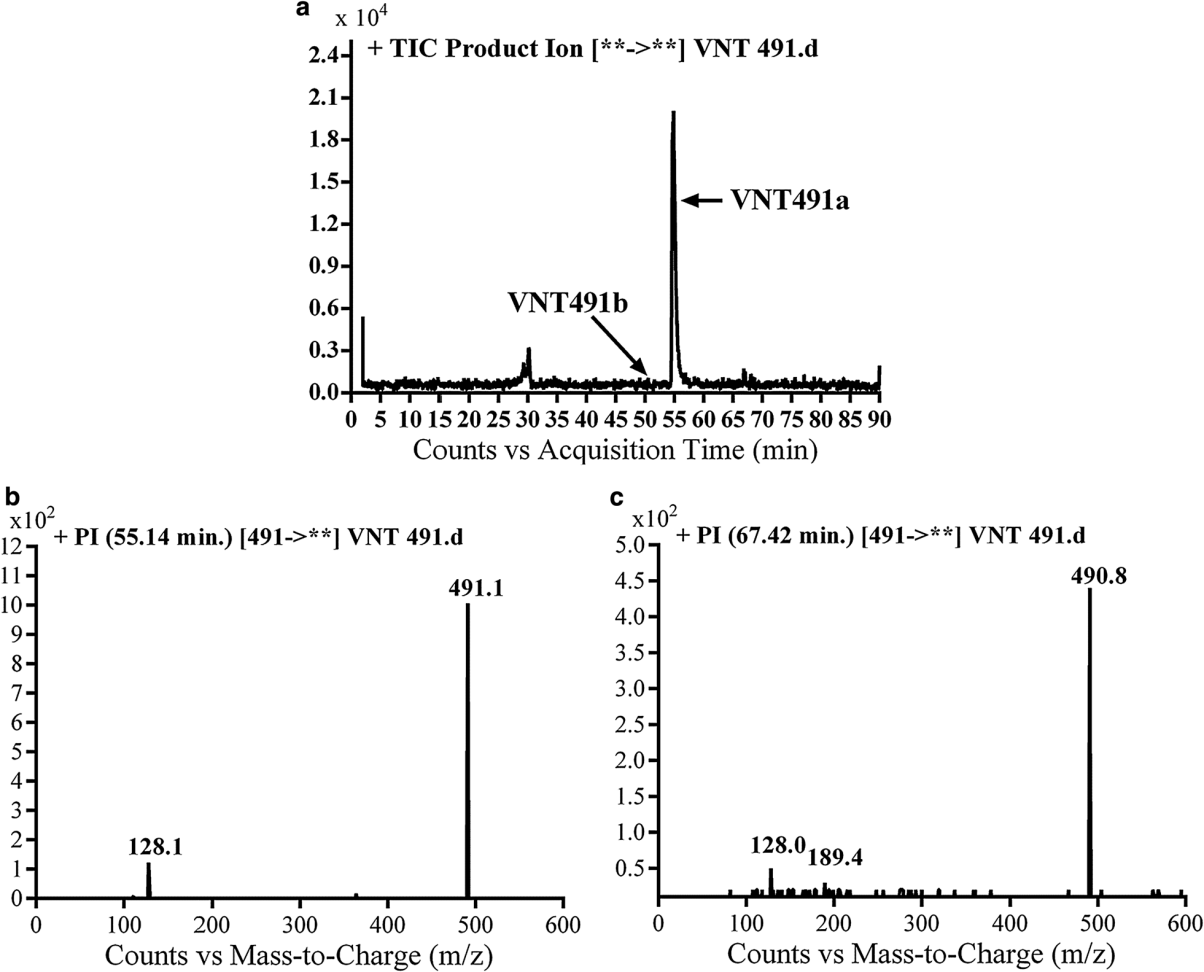
PI chromatogram of MIP at *m/z* 491 showing two peaks: VA491a (57.1 min) and VA491b (67.4 min) (**a**), PI mass spectra of VA491a (**b**) and VA491b at *m/z* 491 (**c**)

VA491a and VA491b metabolites of vandetanib were detected at *m/z* 491 in MS scan mode at t_R_ of 57.1 and 67.4 min, respectively (Fig. 4a). CID of MIPs at *m/z* 491 gave different daughter ions (Fig. 4b, c).

**Scheme 6:**
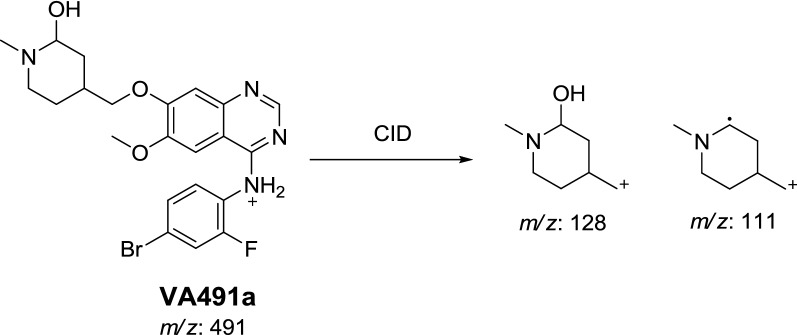
Proposed CID of VA491a

In the case of VA491a, the fragment ion at *m/z* 128 supposed that metabolic reaction was hydroxylation of α-carbon of *N*-methyl piperidine ring which matched with the daughter ion at *m/z* 111 (Scheme 6).

**Scheme 7:**
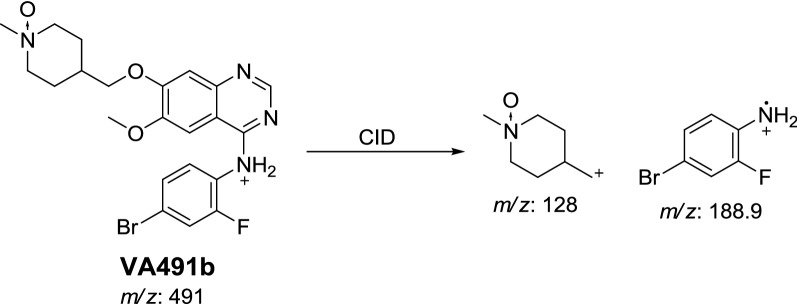
Proposed CID of VA491b

In case of VA491b, the fragment ion at *m/z* 128 proposed that *N*-oxide formation metabolic reaction occurred at *N*-methyl piperidine ring (Scheme 7).

### Characterization of vandetanib reactive metabolites

Extracts of vandetanib in vitro incubations in the presence of 1.0 mM KCN with RLMs were injected into LC-QqQ. Identification of MIPs representing vandetanib cyanide conjugates was performed with mass scan and PI scan for these peaks (Table 4). Four cyanide conjugates were identified, indicating that the *N*-methyl piperidine ring in vandetanib can become bioactivated and then captured by the nucleophile cyanide ion [19].

**Table 4:** Vandetanib cyano conjugates

Code	MS scan	Major fragments	t_R_ (min)	Metabolic pathway	Postulated conjugate composition
VB486	486	389.4, 363.2	71.6	α Cyano addition and *N*-demethylation	V − CH_3_ + CN
VB500a	500a	373.2, 137.1	68.4	α Cyano addition	V + CN
VB500b	500b	456.9, 163.9	76	*N*-demethylation, α oxidation and α Cyano addition	V − CH_3_ + CN + O
VB502	502	484.3, 361.3, 287.4, 203.1	77.1	*N*-demethylation, α hydroxylation and α Cyano addition	V − CH_3_ + CN + OH

#### Identification of VB486 cyano conjugate of vandetanib

**Fig. 5:**
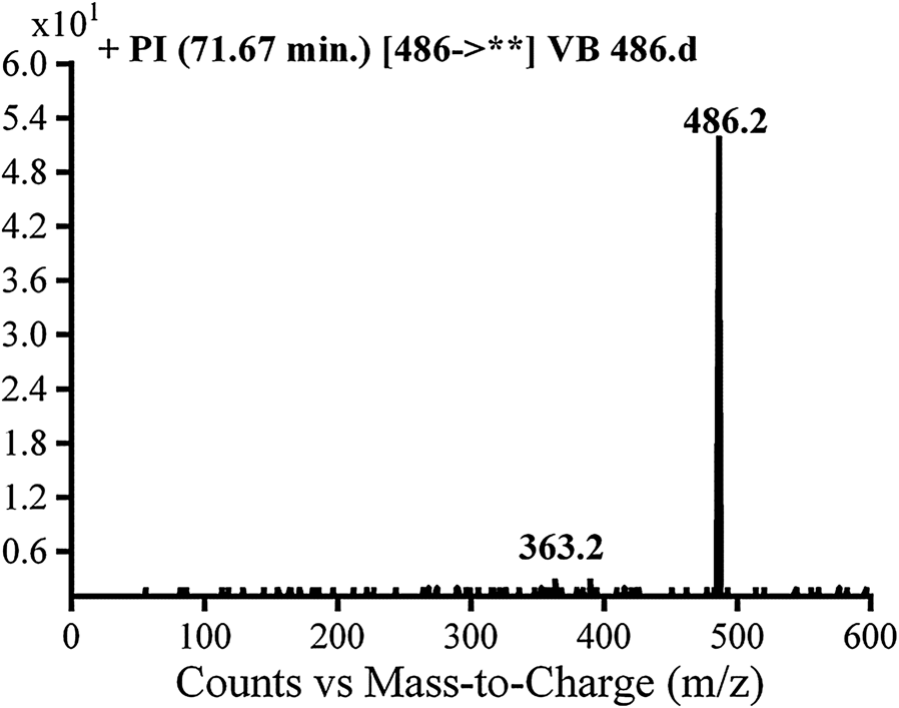
PIs mass spectrum of VB486

**Scheme 8:**
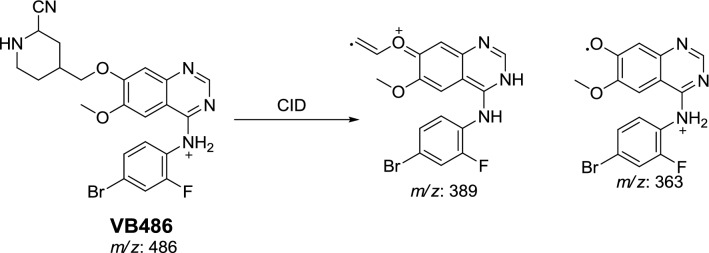
Proposed CID of VB486

VB486 cyano conjugate was detected at *m/z* 486 in MS scan mode with t_R_ of 71.7 min. CID of MIP at *m/z* 486 generates fragment ions at *m/z* 363 and 389 (Fig. 5). The fragment ion at *m/z* 389 proposed cyano group addition to the bio activated α-carbon and *N*-demethylation of piperidine ring. The metabolic pathway in VB486 revealed to α-cyano *N*-demethyl vandetanib (Scheme 8).

#### Identification of VB500a and VB500b cyano conjugates of vandetanib

**Fig. 6:**
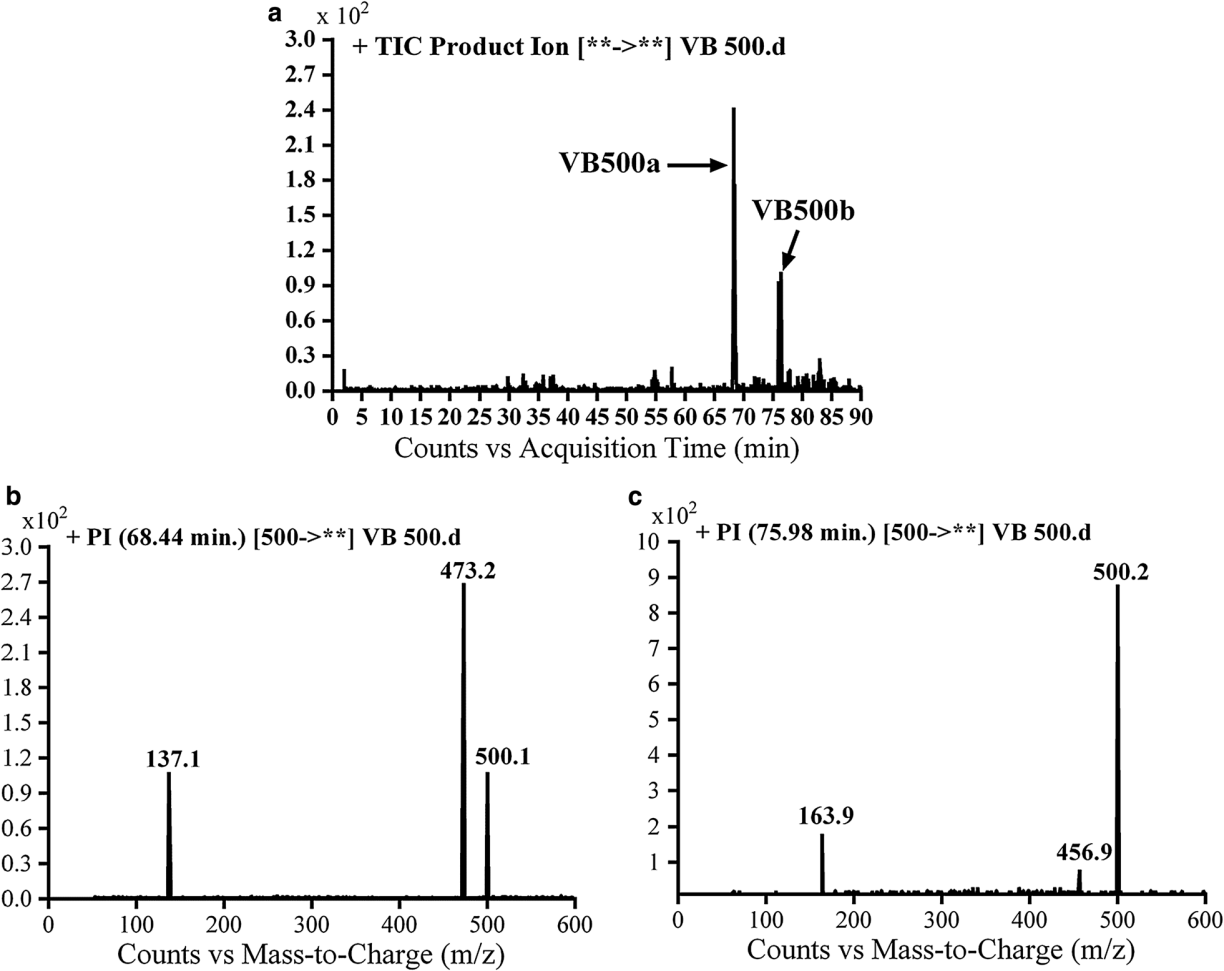
PI chromatogram of MIP at *m/z* 500 showing two peaks: VB500a (68.4 min) and VB500b (75.9 min) (**a**), PI mass spectra of VB500a (**b**) and VB500b (**c**)

VB500a and VB500b cyano conjugates of vandetanib were detected at *m/z* 500 in MS scan mode with t_R_ of 68.4 and 76 min, respectively (Fig. 6a). CID of MIP at *m/z* 500 gave various fragment ions (Fig. 6b, c).

**Scheme 9:**
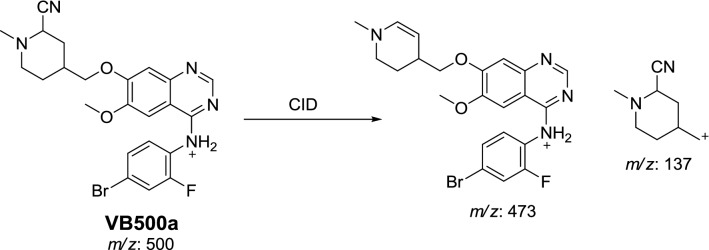
Proposed CID of VB500a

In case of VB500a, the fragment ion at *m/z* 137 proposed that cyano group addition occurred at activated α carbon of the methyl piperidine ring. The other fragment ion at *m/z* 473 represented the cyano group loss (Scheme 9). The metabolic pathway in VB500a revealed to α cyano vandetanib.

**Scheme 10:**
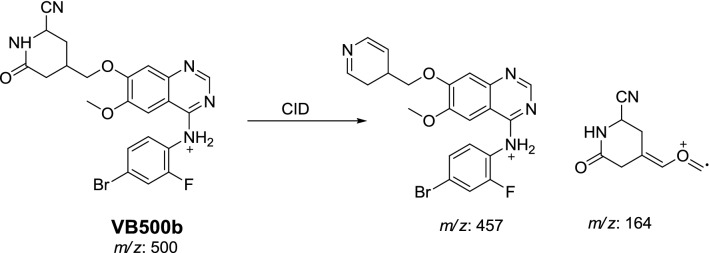
Proposed CID of VB500b

In case of VB500b, fragment ions at *m/z* 164 and *m/z* 457 proposed that α-carbonyl formation, *N*-demethylation and cyano group addition to the activated α carbon (Scheme 10). The metabolic reaction in VB500b revealed to α-cyano α-Keto *N*-demethyl vandetanib.

#### Identification of VB502 cyano conjugate of vandetanib

**Fig. 7:**
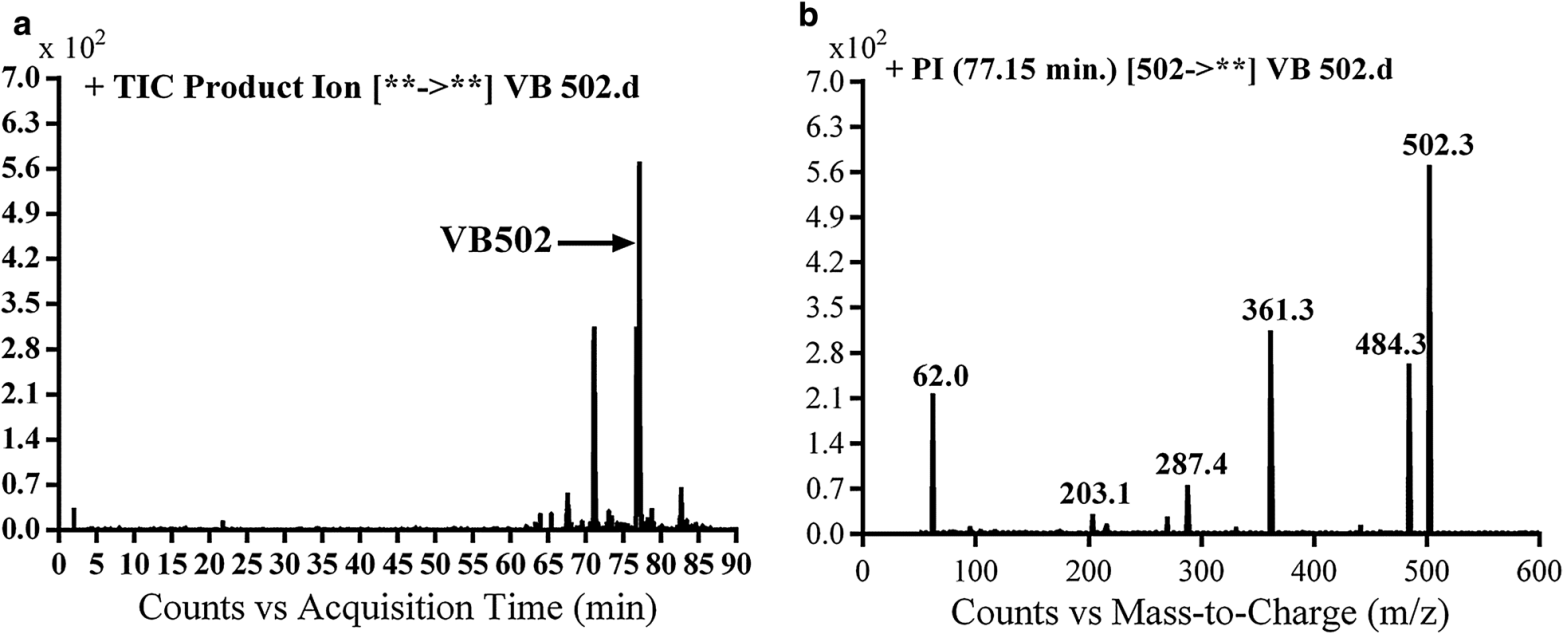
PI chromatogram of MIP at *m/z* 502 showing one peak (VB502) at 77.1 min (**a**), PI mass spectrum of VB502 (**b**)

**Scheme 11:**
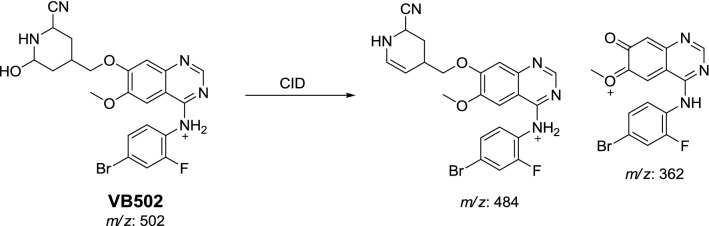
Proposed CID of VB502

VB502 cyano adduct of vandetanib was detected at *m/z* 502 in MS scan mode at t_R_ of 77.1 min (Fig. 7a). CID of MIP at *m/z* 502 generates fragment ions at *m/z* 203, *m/z* 287, *m/z* 362 and *m/z* 484 (Fig. 7b). Daughter ion at *m/z* 362 supposed that all metabolic reactions happened in the piperidine group. Fragment ions at *m/z* 484 and *m/z* 362 proposed that hydroxylation of α carbon, *N*-demethylation of piperidine group and cyano group addition to the activated α-carbon piperidine ring (Scheme 11). The metabolic reaction in VB500b revealed to α-cyano α-hydroxyl vandetanib.

#### Bioactivation mechanism of vandetanib

**Scheme 12:**
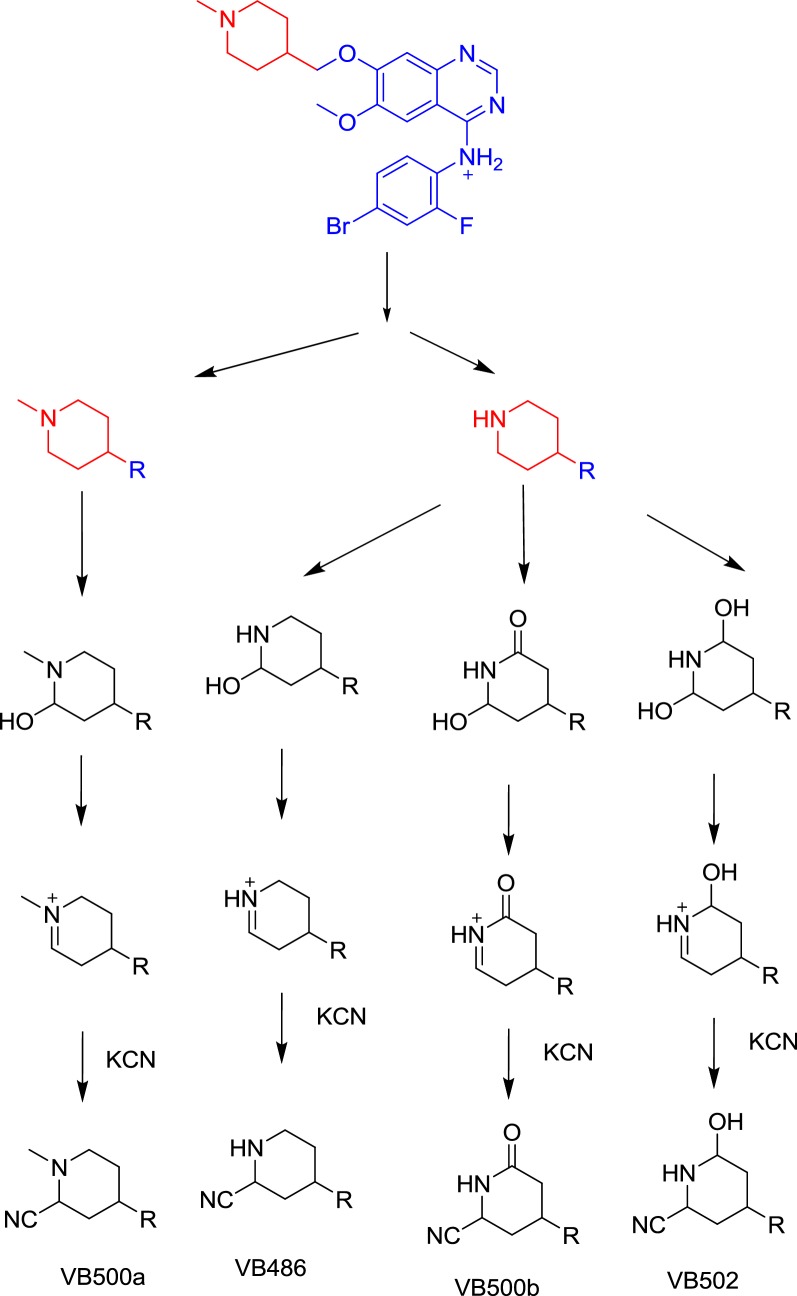
Bioactivation mechanism of piperidine ring of vandetanib

Vandetanib contains cyclic tertiary amine group, *N*-methyl piperidine, that is able to form iminium intermediates which are reactive and can be captured using KCN. The chemical structures of four cyanide conjugates of vandetanib were recognized and bioactivation reactions of the *N*-methyl piperidine ring were explained as shown in Scheme 12. The *N*-methyl piperidine ring in vandetanib underwent P450-catalyzed oxidation and or hydroxylation and subsequent dehydration forming imine and imine-carbonyl intermediates (α,β-unsaturated) which was trapped by KCN to form stable conjugate that was characterized and detected in the tandem mass spectrometry detector [19, 20, 24, 25].

### Identification of vandetanib in vivo metabolites

PI mass spectra comparison between control urine samples with urine extracts as well as PI comparison of vandetanib and proposed metabolites (Table 3) permitted the identification of four in vivo phase I and one phase II metabolites. Metabolic reactions for in vivo phase I metabolites were proposed to be *N*-oxide formation, *N*-demethylation and α-hydroxylation while for phase II metabolites were the result of N-conjugation of vandetanib with glucuronic acid. In vivo vandetanib phase I metabolites are previously mentioned in in vitro vandetanib phase I metabolism.

#### Excretion of vandetanib and its in vivo metabolites in rat urine

**Fig. 8:**
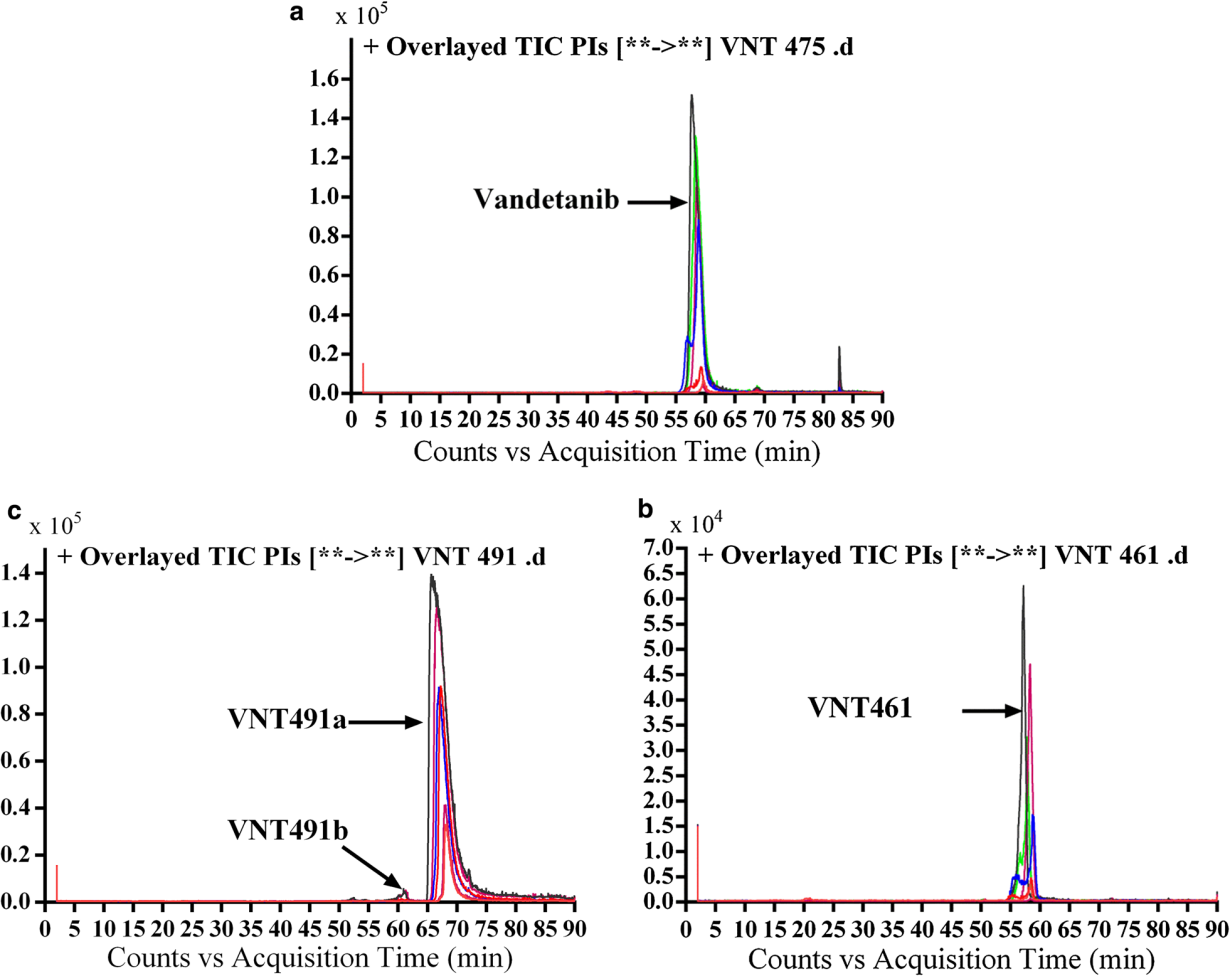
Overlayed PI chromatograms of vandetanib (58.3 min) and VC475 (68.6 min) (**a**), VC461 (57.2 min) (**b**) and VC491a (56.5 min) and VC491b (67.0 min) (**c**)

Part of vandetanib oral dose was excreted unchanged in rat urine. Vandetanib was detected at *m/z* 475 in MS scan spectrum. Excretion of vandetanib and its in vivo phase I metabolites in urine were noticed after 6 h of dosing. Comparative concentration of vandetanib was high after 6 h and began to increase by time and reach maximum at 24 h and begin to decrease until almost disappeared after 120 h from dosing as indicated in the overlayed PI chromatograms for vandetanib and its in vivo phase I metabolites (Fig. 8). Comparison of vandetanib PIs with proposed peaks permitted the identification of metabolic changes in the found in vivo metabolites. All in vivo metabolites are similar to in vitro metabolites and the disappearance of α oxidation metabolic reaction in the in vivo metabolism.

#### Phase II vandetanib in vivo metabolites: glucuronic acid conjugates

**Fig. 9:**
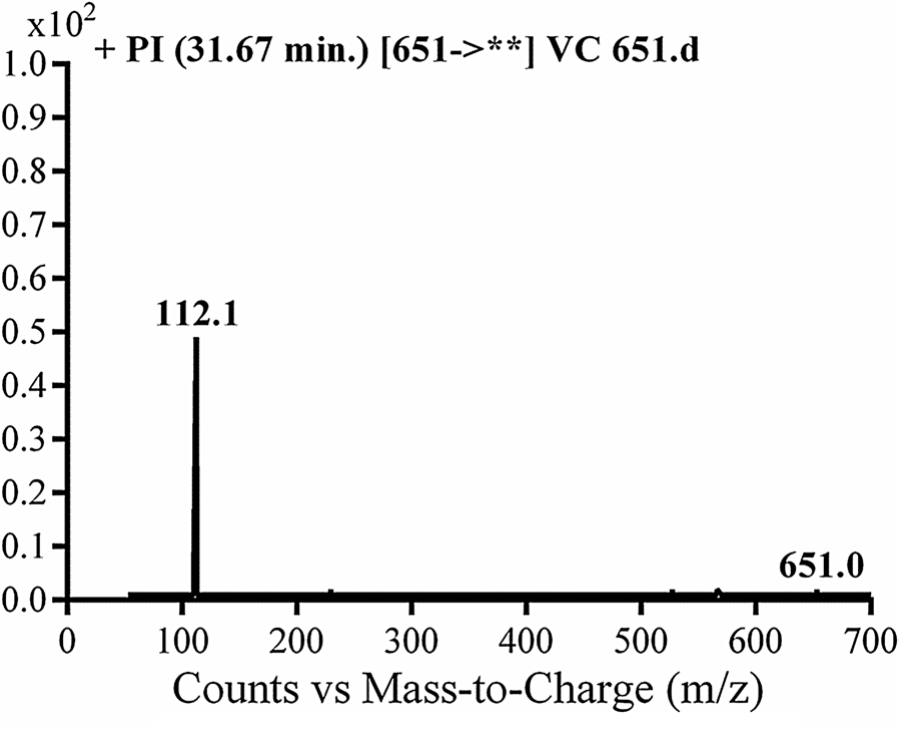
PI mass spectrum of VC651

**Scheme 13:**
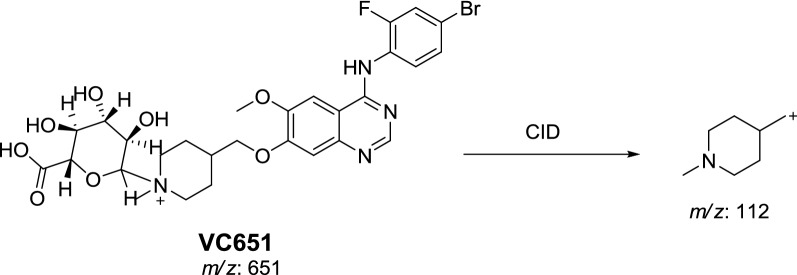
Proposed CID of VC651

In vivo phase II metabolic reaction was direct conjugation of vandetanib with glucuronic acid. VC651 was located in the aqueous layer in a very small concentration in comparison to in vivo phase I metabolites. Excretion of in vivo phase II metabolites in urine was noticed after 24 h of rat dosing and vanished rapidly after 48 h of rat dosing. VC651 was detected at *m/z* 651 in MS scan spectrum of the aqueous layer urine extract. PI scan for VC651 at 31.7 min gave fragment ions at *m/z* 112 (Fig. 9). VC651 was proposed to be the result of was direct conjugation of glucuronic acid with vandetanib (Scheme 13).

## Conclusions

**Fig. 10:**
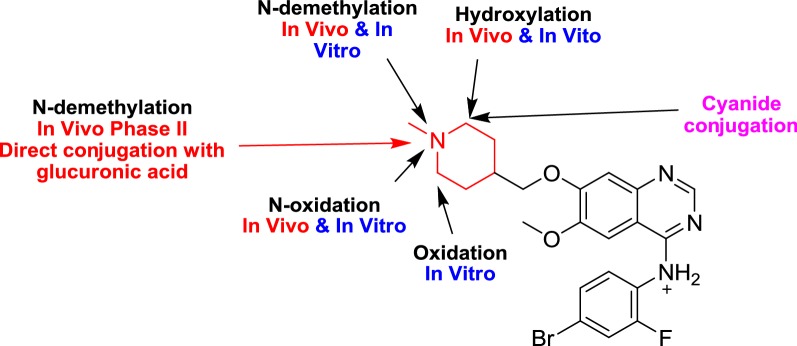
Proposed in vitro metabolites, in vivo metabolites and cyano conjugates of vandetanib

Four in vivo phase I, one in vivo phase II metabolites and six in vitro phase I were found for vandetanib. Phase I metabolic pathways for vandetanib were *N*-demethylation, *N*-oxide formation, α-carbonyl formation and α-hydroxylation. All phase I metabolic pathways happened in *N*-methyl piperidine of vandetanib (Fig. 10). Four cyano adducts were characterized. All metabolic and bioactivation reactions occurred in the *N*-methyl piperidine part which causes toxicity and instability of vandetanib (Additional file 1).

## Additional file

**Additional file 1: Figure S1.** PI chromatograms of molecular ions at *m/z* 475 of organic extract of control urine sample taken before masitinib dosing showing no peaks at 58.3 min. and 68.6 min. **Figure S2.** PI chromatograms of molecular ions at *m/z* 461 of organic extract of control urine sample taken before vandetanib dosing showing no peak at 27.9 min. **Figure S3.** PI chromatograms of molecular ions at *m/z* 491 of organic extract of control urine sample taken before vandetanib dosing showing no peak at 56.5 and 67.0 min. **Figure S4.** Product ion chromatogram of molecular ion peak at *m/z* 475 showing two peaks: VC491a (56.5 min) and VC491b (67.0 min). **Figure S5.** PI mass spectrum of molecular ion peak (vandetanib) at *m/z* 475. **Figure S6**: PI mass spectrum of molecular ion peak (VC475) at *m/z* 475. **Figure S7.** Product ion chromatogram of molecular ion peak at *m/z* 461 showing one peak: VC461 (57.2 min). **Figure S8.** PI mass spectrum of molecular ion peak (VC461) at *m/z* 461. **Figure S9.** Product ion chromatogram of molecular ion peak at *m/z* 491 showing two peaks: VC491a (56.5 min) and VC491b (67.0 min). **Figure S10.** PI mass spectrum of molecular ion peak (VC491a) at *m/z* 491. **Figure S11.** PI mass spectrum of molecular ion peak (VC491b) at m/z 491.
