# Supplementary material for: Identification and characterization of in vivo, in vitro and reactive metabolites of vandetanib using LC–ESI–MS/MS
Source: Chem Cent J. 2018 Sep 24;12:99. doi: 10.1186/s13065-018-0467-5 (PMC6768145; doi:10.1186/s13065-018-0467-5)
Supplement: Supplementary file 1 — Additional file 1: Figure S1. PI chromatograms of molecular ions at m/z 475 of organic extract of control urine sample taken before masitinib dosing showing no peaks at 58.3 min. and 68.6 min. Figure S2. PI chromatograms of molecular ions at m/z 461 of organic extract of control urine sample taken before vandetanib dosing showing no peak at 27.9 min. Figure S3. PI chromatograms of molecular ions at m/z 491 of organic extract of control urine sample taken before vandetanib dosing showing no peak at 56.5 and 67.0 min. Figure S4. Product ion chromatogram of molecular ion peak at m/z 475 showing two peaks: VC491a (56.5 min) and VC491b (67.0 min). Figure S5. PI mass spectrum of molecular ion peak (vandetanib) at m/z 475. Figure S6: PI mass spectrum of molecular ion peak (VC475) at m/z 475. Figure S7. Product ion chromatogram of molecular ion peak at m/z 461 showing one peak: VC461 (57.2 min). Figure S8. PI mass spectrum of molecular ion peak (VC461) at m/z 461. Figure S9. Product ion chromatogram of molecular ion peak at m/z 491 showing two peaks: VC491a (56.5 min) and VC491b (67.0 min). Figure S10. PI mass spectrum of molecular ion peak (VC491a) at m/z 491. Figure S11. PI mass spectrum of molecular ion peak (VC491b) at m/z 491. [file 13065_2018_467_MOESM1_ESM.docx]

**Additional Figures**

**1-Rat urine control samples organic layer extract**

**
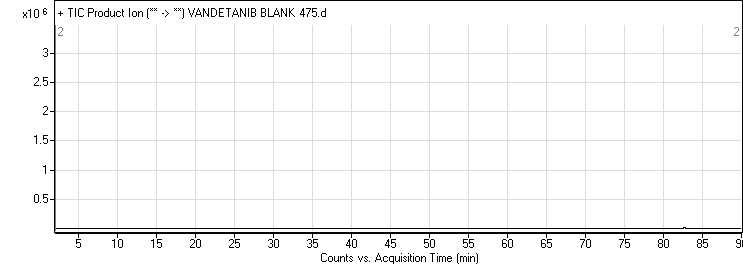
**

**Figure S1**: PI chromatograms of molecular ions at *m/z* 475 of organic extract of control urine sample taken before masitinib dosing showing no peaks at 58.3 min. and 68.6 min.

**
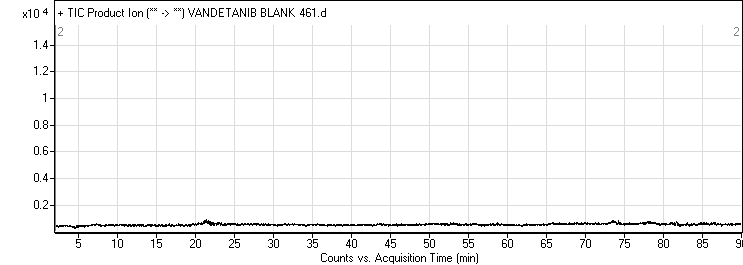
**

**Figure S2**: PI chromatograms of molecular ions at *m/z* 461 of organic extract of control urine sample taken before vandetanib dosing showing no peak at 27.9 min.

**
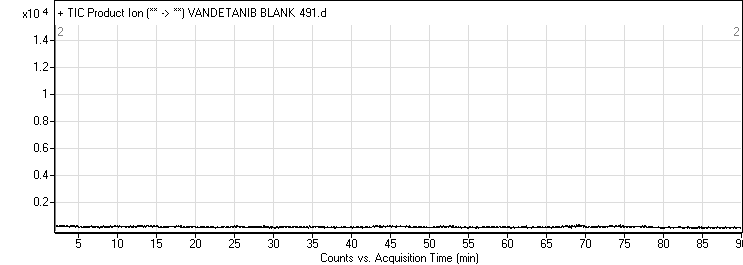
**

**Figure S3**: PI chromatograms of molecular ions at *m/z* 491 of organic extract of control urine sample taken before vandetanib dosing showing no peak at 56.5 and 67.0 min.


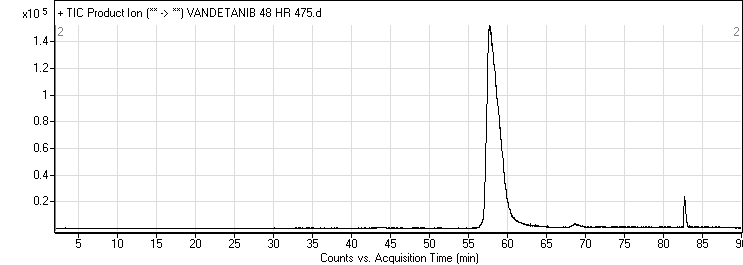


**Figure S4:** Product ion chromatogram of molecular ion peak at *m/z* 475 showing two peaks: VC491a (56.5 min) and VC491b (67.0 min).


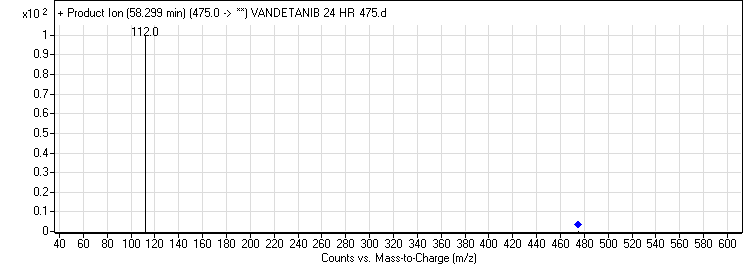


**Figure S5**: PI mass spectrum of molecular ion peak (vandetanib) at *m/z* 475.


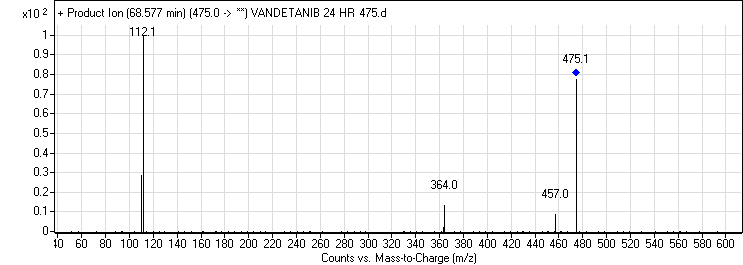


**Figure S6**: PI mass spectrum of molecular ion peak (VC475) at *m/z* 475.


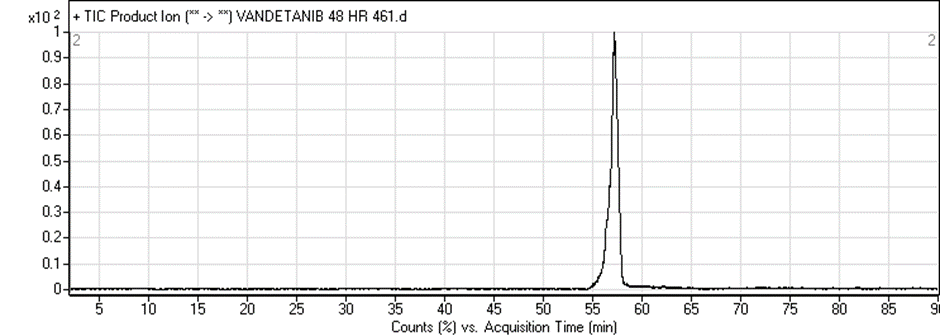


**Figure S7:** Product ion chromatogram of molecular ion peak at *m/z* 461 showing one peak: VC461 (57.2 min).

**
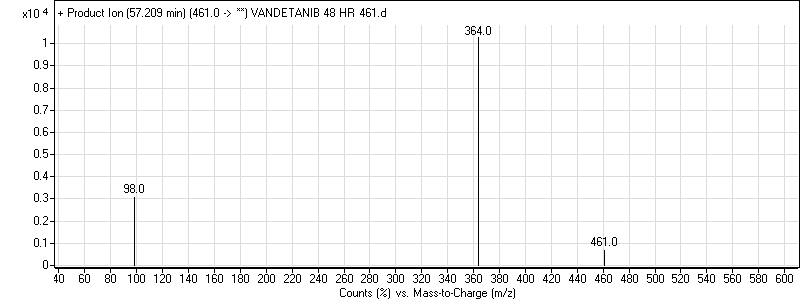
**

**Figure S8**: PI mass spectrum of molecular ion peak (VC461) at *m/z* 461.


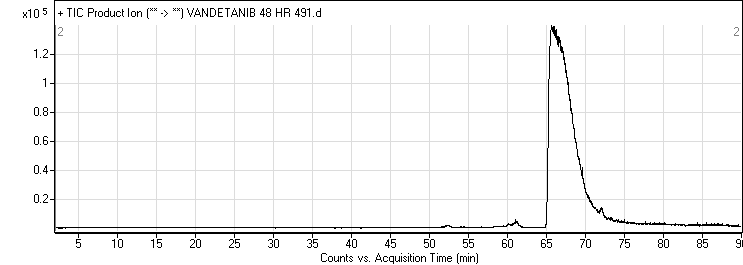


**Figure S9:** Product ion chromatogram of molecular ion peak at *m/z* 491 showing two peaks: VC491a (56.5 min) and VC491b (67.0 min).


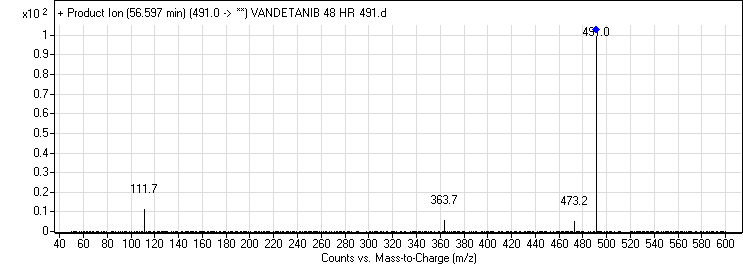


**Figure S10**: PI mass spectrum of molecular ion peak (VC491a) at *m/z* 491.


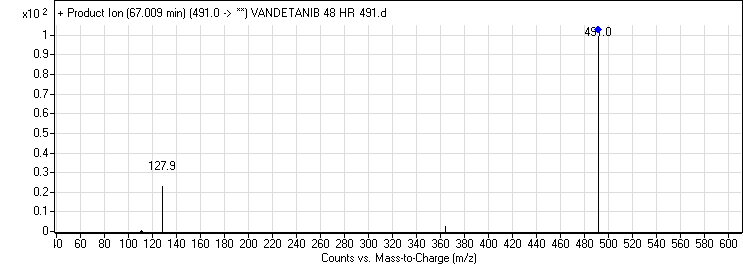


**Figure S11**: PI mass spectrum of molecular ion peak (VC491b) at m/z 491.
